# Variable predicted pathogenic mechanisms for novel MECP2 variants in RTT patients

**DOI:** 10.1186/s43141-022-00305-8

**Published:** 2022-03-11

**Authors:** Wessam E. Sharaf-Eldin, Mahmoud Y. Issa, Maha S. Zaki, Ayman Kilany, Alaaeldin G. Fayez

**Affiliations:** 1 Medical Molecular Genetics Department, Human Genetics and Genome Research institute, National Research Centre, Cairo, 12311 Egypt; 2 Clinical Genetics Department, Human Genetics and Genome Research institute, National Research Centre, Cairo, Egypt; 3 Department of Research on Children with Special Needs, Medical Research Institute, National Research Centre, Cairo, Egypt; 4 Molecular Genetics and Enzymology Department, Human Genetics and Genome Research Institute, National Research Centre, Cairo, Egypt

**Keywords:** MECP2, Rett syndrome, Missense mutation, In silico analysis

## Abstract

**Background:**

Methyl CpG binding protein 2 (MeCP2) is essential for the normal function of mature neurons. Mutations in the MECP2 gene are the main cause of Rett syndrome (RTT). Gene mutations have been identified throughout the gene and the mutation effect is mainly correlated with its type and location.

**Methods:**

In this study, a series of in silico algorithms were applied for analyzing the functional consequences of 3 novel gene missense mutations (D121A, S359Y, and P403S) and a rarely reported one with suspicious effect (R133H) on RettBASE. Besides, a ROC curve analysis was performed to investigate the critical factors affecting variant pathogenicity.

**Results:**

(1) The ROC curve analysis for a retrieved set of MeCP2 variants showed that physicochemical characters do not significantly affect variant pathogenicity; (2) PREM PDI tool revealed that both D121A and R133H mainly contribute to disease progression via reducing MeCP2 affinity to DNA; (3) GPS v5.0 software indicated that P403S may correlate with altered protein phosphorylation; however, no defective protein interaction has been already documented. (4) The applied computational algorithms failed to explore any informative pathogenic mechanism for the S359Y variant.

**Conclusion:**

The conducted approach might provide an efficient prediction model for the effect of MECP2 variants that are located in MBD and CTD.

**Supplementary Information:**

The online version contains supplementary material available at 10.1186/s43141-022-00305-8.

## Background

Rett syndrome (RTT) (RTT, OMIM #312750) is a severe neurodevelopmental disorder that mainly affects females. It represents the second most common cause of intellectual disability in females after Down syndrome with an estimated incidence of one in 10,000 female births [1]. The classic disease form is characterized by a period of normal psychomotor development during the first 6 to 18 months of life, followed by loss of already acquired functions, such as motor ability, communication skills, and purposeful hand movement. Additional key features include stereotypic hand movements, acquired microcephaly, seizures, and breathing difficulties [2]. Patients, lacking one or more of the disorder’s main characteristics, defined as atypical or variant RTT cases comprise five clinical entities, namely preserved speech, late regression, forme fruste congenital, and early-onset seizure variants [3]. Mutations in the X-linked gene encoding methyl CpG binding protein 2 (MECP2) are the master cause of RTT [4]. However, pathogenic mutations in many other genes (< 70) including CDKL5, FOXG1, Netrin G1, MEF2C, and SCN1A have been detected in patients diagnosed with RTT without disease-causing MECP2 mutation [5]. It is noteworthy that RTT is frequently misdiagnosed, especially by primary care physicians. Therefore, molecular testing and functional analysis (computationally or experimentally) for novel missense variations are strongly required to provide precise genetic counseling [6]. Based on the spectrum of RTT variants, multiple hypotheses were formulated to address the influence of mutations on phenotype based on their type and location. Generally, patients with either missense mutations or deletions within the hotspot C-terminal region (c.1056–c.1165) tend to present milder phenotype [7]. However, a staightforward relationship between clinical phenotype and MECP2 mutations does not exist [8]. Expression mosaicism between the normal and the mutant alleles due to X-chromosome inactivation (XCI) is a key contributor to phenotypic variability [9]. Moreover, genetic modifiers [10] and environmental factors [11] might also contribute to clinical severity.

MECP2 gene encodes the MeCP2 protein consisting of 486 amino acids. The main functional protein domains are the methylated DNA binding domain (MBD) (p.77–p.163) and the transcriptional repression domain (TRD) (p. 206–p.310) mediating protein binding to methylated DNA and the consequent transcriptional regulation, respectively. Within the TRD, there is a nuclear localization signal (NLS). The protein also contains the N-terminal domain (NTD), intervening domain (ID), and C-terminal region that contains two domains (CTD α and β) [12].

Most missense mutations causing RTT affect MBD and TRD. However, it has been revealed that some mutations that fall outside these domains can impact the protein activity [13]. Attention is only confined to variants absent in parental DNA. Importantly, it has been found that other domains (ID, CTDα, and CTDβ) affect DNA interaction via direct and indirect ways [14].

The main domain characteristic is its distinct conformation that enables structure prediction and contributes to whole protein modeling. MBD is the best characterized MeCP2 domain whose structured images have been released either free in solution [15] or bound to methylated DNA [16]. In this context, it is relatively conclusive to predict the effect of variants within this region. However, more efforts are still needed to investigate the influence of other sequence variations escaping MBD. The present study was mainly aimed to explore the impact of certain MeCP2 missense variants inside and outside MBD and illustrate their possible molecular pathogenic mechanisms, using a variety of predictive computational tools.

## Methods

### Detection of variants

Studied variants were detected during molecular analysis of MECP2 gene in females with provisional diagnosis of Rett syndrome (RTT) as previously mentioned in Sharaf-Eldin et al. [17]. Data for human MECP2 gene sequence and variants were collected from NCBI (http://www.ncbi.nlm.nih.gov/), ensemble (https://www.ensembl.org/index.html), RettBASE variation database (http://mecp2.chw.edu.au/index.shtml#mutations), and ClinVar (https://www.ncbi.nlm.nih.gov/clinvar/).

### ROC curve analysis

A receiver operating characteristic (ROC) curve analysis was performed to determine whether (1) the difference of physicochemical characteristics between wild and mutant amino acids could significantly influence the effect of missense mutations and (2) physicochemical-based effect can be formulated. Subsequent to individual parameter testing, a stepwise combined analysis was carried out to investigate if any parameter composite may earn higher potential. Studied physicochemical characteristics were polarity, charge, and hydrocarbon type. In addition, annotated location was also involved. Analysis was applied for variants with known clinical significance on ClinVar (https://www.ncbi.nlm.nih.gov/clinvar/) in addition to our studied variants with a total number of 45 gene variations (Supplementary table 1). Statistical analyses were conducted using the Statistical Package for the Social Sciences (SPSS, version 18.0; IBM Corp., Chicago, USA, 2009), and a *P* value of <0.05 was considered statistically significant.

### Bioinformatics analysis

rsIDs of the reported missense variants were extracted from the dbSNP (https://www.ncbi.nlm.nih.gov/snp/) and ClinVar (https://www.ncbi.nlm.nih.gov/clinvar/) databases. rsIDs were used as query sequences to explore the possible effect of the studied variants.

The used pathogenicity prediction tools can be classified into:

1. Conservation and amino acid substitution matrices-dependent pathogenicity prediction tools: VEP (https://www.ensembl.org/Tools/VEP), MutationTaster2 (http://www.mutationtaster.org/), AGVGD (http://agvgd.hci.utah.edu/agvgd_input.php), and Blosum62 (amino acid substitution matrices from protein blocks)
2. Gene Ontology GO-dependent pathogenicity prediction tool: SNPs&GO (https://snps-and-go.biocomp.unibo.it/snps-and-go/index.html)
3. Protein structural and functional site-dependent pathogenicity prediction tools: MutPred2 (http://mutpred.mutdb.org/), PROVEAN (http://provean.jcvi.org/index.php), PolyPhen2 (http://genetics.bwh.harvard.edu/pph2/) to explore the variant effects on the protein structure, Swiss PDB Viewer to visualize protein 3D structure (energy minimization was carried out using GROMOS96), BIOVIA discovery studio visualizer v19.1.0 to annotate non-bond interactions, Protein Plus server to explore MECP2-methylated DNA topology, and PREM PDI to calculate binding affinity change (∆∆G)
4. Splicing modification-dependent pathogenicity prediction tool: ESEfinder v3.0
5. Secondary mRNA folding-dependent pathogenicity prediction tool: Mfoldserver
6. Post-transcriptional modification-dependent pathogenicity prediction tool: GPS v5.0

## Results

### Detected variants

Four MECP2 variants (D121A, R133H, S359Y, and P403S) were illustrated in this report. They are all novel except R133H which is rarely reported in RTT patients (frequency: 0.17%) with unknown pathogenicity [4]. P403S was found in a girl with atypical RTT. However, others were reported in patients with typical RTT. Clinical manifestations and neuroimaging of patients are summarized in supplementary table 2. Importantly, the S359Y mutation was found in combination with one of the most common RTT mutations, R168X.

### ROC curve analysis

According to our ROC curve analysis, variant location is the only significant factor in expecting variation pathogenicity with the area under the curve (AUC) of 0.871. Where MBD and TRD mutations are most likely to have a pathogenic effect, regardless of the physicochemical characteristics of wild type and mutant amino acids. Results of ROC curve analysis are illustrated in supplementary table 3.

### Computational prediction for the effect of detected variants

Both R133H and D121A gave positive pathogenic scores across protein structural and functional site-dependent pathogenicity prediction tools including PROVEAN, PolyPhen2, and MutPred2 as listed in Table 1. Therefore, the pathogenic effect of both R133H and D121A might depend on altering the protein conformation. This hypothesis was subsequently examined in the following analysis via more advanced exploration tools. However, it is most likely that S359Y and P403S are benign gene variations.

**Table 1:** Predicting the effect of detected variants

Tool/database	C1076A (S359Y) Ser359Tyr TCC → TAC	C1207T (P403S) Pro403Ser CCT → TCT	G398A (R133H) Arg133His CGC → CAC	A362C (D121A) Asp121Ala GAT → GCT
MutationTaster2 (score)^a^	Disease causing (155)	Disease causing (74)	Disease causing (29)	Disease causing (126)
PROVEAN^b^	Neutral (−1.189)	Neutral (−0.315)	Deleterious (−4.996)	Deleterious (−7.838)
PolyPhen2 (score)^c^	Benign (0.085) Sensitivity: 0.93 Specificity: 0.85	Benign (0.141) Sensitivity: 0.92 Specificity: 0.86	Probably damaging (1.000) Sensitivity: 0.00 Specificity: 1.00	Probably damaging (1.000) Sensitivity: 0.00 Specificity: 1.00
AGVGD^d^	Class C65	Class C65	Class C25	Class C65
SNPs&GO (reliability index)^e^	Neutral (8)	Neutral (9)	Disease-related (6)	Disease-related (5)
MutPred2 software (probability)^f^	Non-deleterious (0.197)	Non-deleterious (0.106)	Deleterious (0.721).	Deleterious (0.860)
Blosum62^g^	−2	−1	0	−2
ESEfinder 2.0^h^	Insignificant	Insignificant	Insignificant	Insignificant

^a^MutationTaster score may range from 0.0 (polymorphism) to 215 (disease causing)

^b^Variants with a score equal to or below −2.5 are considered “deleterious” and variants with a score above −2.5 are considered “neutral”

^c^PolyPhen2 score ranges from 0.0 (tolerated) to 1.0 (deleterious)

^d^AGVGD classes: C65 (most likely to interfere with function), C55, C45, C35,C25, 15, C0 (least likely to interfere with function)

^e^The SNPs&GO reliability index ranges from 1 to 10 (with 10 being the highest)

^f^MUTPRED predictions are listed as “Deleterious” if the score is >0.5

^g^In the Blosum62 matrix, a positive score implies that substitution is more likely than any random substitution and vice versa

^h^ESEfinder adjusted to score the best hit in each sequence

### Prediction of the MeCP2 protein model

**Fig. 1:**
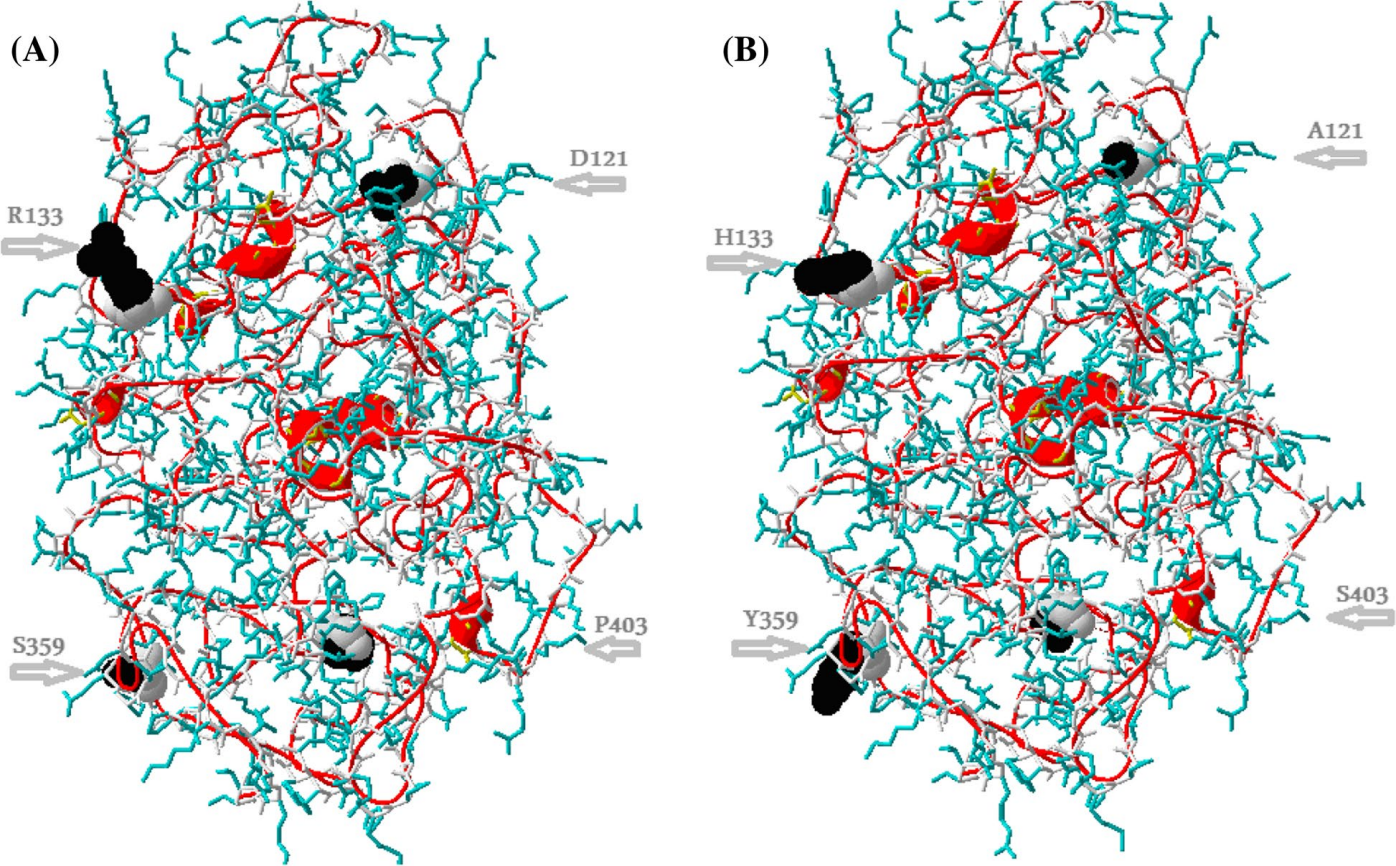
The whole MeCP2 protein model using Phyre^2^ server with localized annotated detected variants. **A** Wild MeCP2 and **B** mutant MeCP2. The wild and mutant amino acids are represented by volumed ribbon configuration, and the gray arrows indicate the outer position of the detected variants

Modeling of whole MeCP2 protein was achieved using physical-chemical properties of unsolved amino acids (ab initio prediction approach) and solved MeCP2 MBD (homology modeling approach). A combination between the two approaches is necessary, since the ab initio prediction approach only gives ultimately insignificant information. The wild and mutant MeCP2 protein models are shown in Fig. 1, and the quality parameters of the structured MeCP2 model are reported in Table 2. The physiochemical characteristics of wild and mutant amino acids are shown in Table 3.

**Table 2:** Quality parameters of the structured MeCP2 model using Swiss PDB Viewer 4.1.0 program

Quality parameters	D121	R133	S359	P403
Residues with conformational angles lie outside allowed regions	**-**	**-**	**-**	**+**
Residues deviating from Trans peptide bonds	**-**	**-**	**+**	**+**
Residues with protein problems	-	-	Buried side chain^a^	-
Residues according to its threading energy	Within normal range	Within normal range	Within normal range	Within normal range

(-) means score lies in allowed range, but (+) means score lies out allowed range

^a^Residues that could make H bonds but do not

**Table 3:** Physical-chemical properties of wild and mutant amino acids

		Charge	Volume	Polarity
**D121A** (in MBD)	Wild	Negative	Larger	Polar
	Mutant	Neutral	Smaller	Nonpolar
**R133H** (in MBD)	Wild	Positive	Larger	Basic
	Mutant	Neutral	Smaller	Basic
**S359Y** (in CTD)	Wild	Neutral	Smaller	Polar
	Mutant	Neutral	Larger	Polar
**P403S** (in CTD)	Wild	Neutral	–	Nonpolar
	Mutant	Neutral	–	Polar

### Both Arg111 and Arg133 finger residues are rigidified by salt bridge bonds with D121

In the current research, the PDB ID 6OGK structure model was mainly utilized due to its highest resolution (1.65Å) in comparison to other registered X-ray crystal PDB structures.

**Fig. 2:**
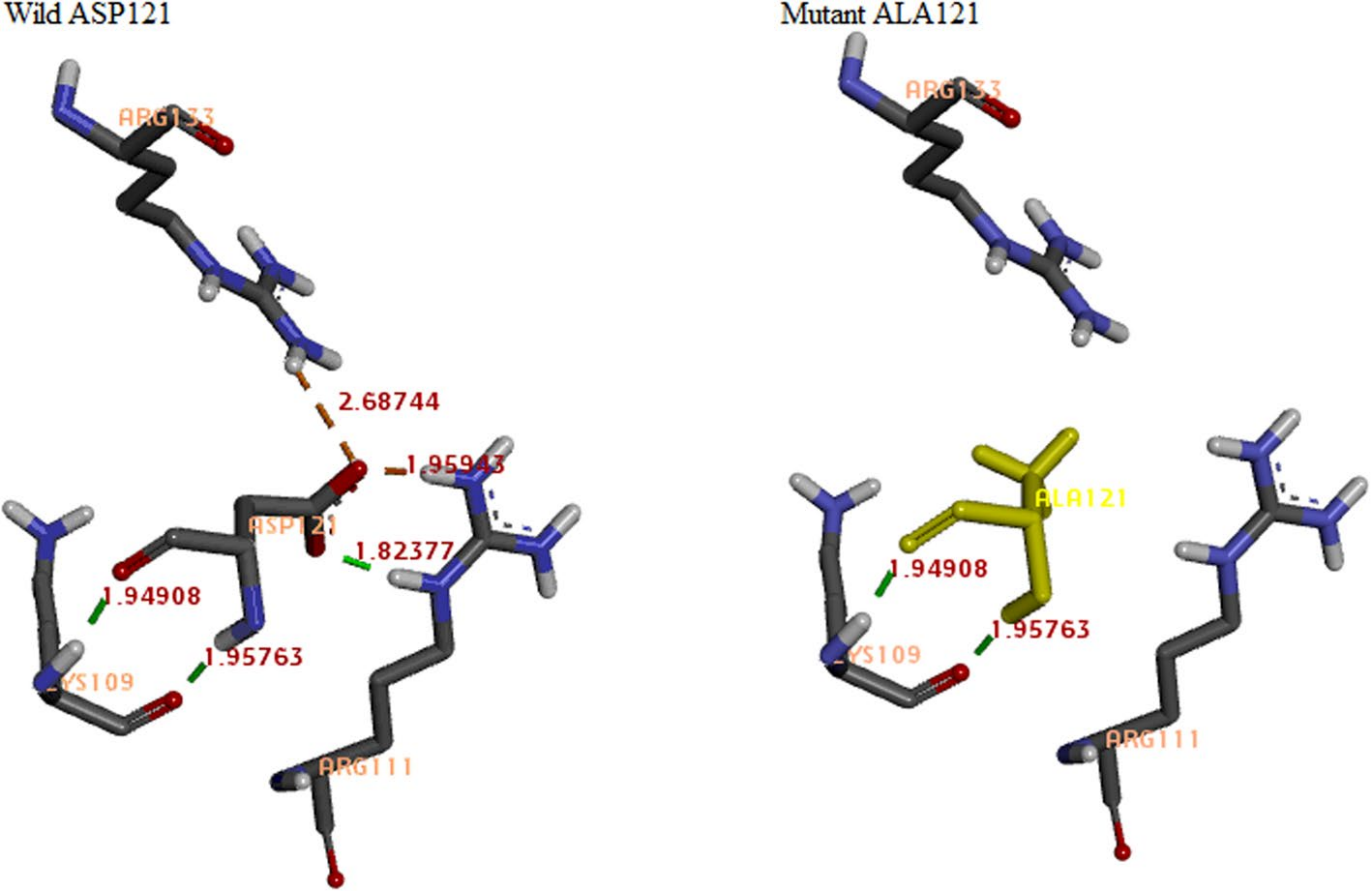
The D121A protein model based on PDB ID 6OGK showing different bonding capacities with neighboring residues. Green dot lines represent hydrogen bonds and purple dot lines represent salt bridges with the corresponding bond distances. Energy minimization was done using GROMOS96 implementation in Swiss PDB Viewer under the default settings [total energy=−3366.514 kJ/mol]

According to PDB ID 6OGK model-based Proteins Plus analysis, Structure-Based Modeling Support Server, Swiss PDB Viewer, and BIOVIA discovery studio visualizer [18], the main hydrocarbon chain of Asp121 forms two strong H bonds with Lys109. However, its side chain forms one strong hydrogen (H) bond with Arg111 and two salt bridges with Arg111 and Arg133 as shown in Table 4 and Fig. 2. It is noteworthy that salt bridges play an important role for the stability and rigidity of native protein structure and conformation. Detected D121A variant converts aspartic acid residue to alanine abolishing salt bridge formation with Arg111 and Arg133 and inhibiting H bonding with Arg111. Such different bonding capacities can lead to increased plasticity of Arg111 and Arg133 finger residues which are essential for MBD-DNA binding. Therefore, it is strongly predicted that D121A affects MeCP2 conformation and results in reduced protein affinity for DNA.

**Table 4:** Differential non-bond interaction of D121A variant between wild and mutant states

Wild [D121]	Mutant [A121]	Non-bond interaction type	Distance (A°)^**a**^
ARG111:ASP121	---	Salt bridge	1.95
ARG133:ASP121	---	Salt bridge	2.69
LYS109:ASP121	LYS109:ASP121	Strong H. bond	1.95
ARG111:ASP121	---	Strong H. bond	1.82
ASP121:LYS109	ASP121:LYS109	Strong H. bond	1.96

^a^Under steric bump; VDW fraction = 0.70; max. dist. of strong H. bond = 3.40; max. dist. of weak H. bonds = 3.8; max. dist. of the salt bridge = 4.0

### D121A and R133H affect the binding affinity of MDB-DNA complex within 5′-GTG-3′ and 5′-mCAC-3′ sequences

To extend the pathogenicity interpretation of D121A and R133H, we used the PREM PDI tool to calculate the effect of these point variants on the binding affinity change (∆∆G) and it was revealed that both D121A and R133H led to significant ∆∆G and lower thermostability of the MBD-DNA complex as shown in Table 5.

**Table 5:** ∆∆G of D121A and R133H

Variant	∆∆G kcal mol^−1a^		
	Unmethylated DNA strand	Methylated DNA strand	Both DNA strands.
Asp121Ala	**1.17**	0.48	0.40
Arg133His	0.93	1.08	**1.26**

^a^ Calculated by PREM PDI tool, deleterious threshold point is 1.10 kcal mol^−1^ corresponding to dataset ROC analysis

**Fig. 3:**
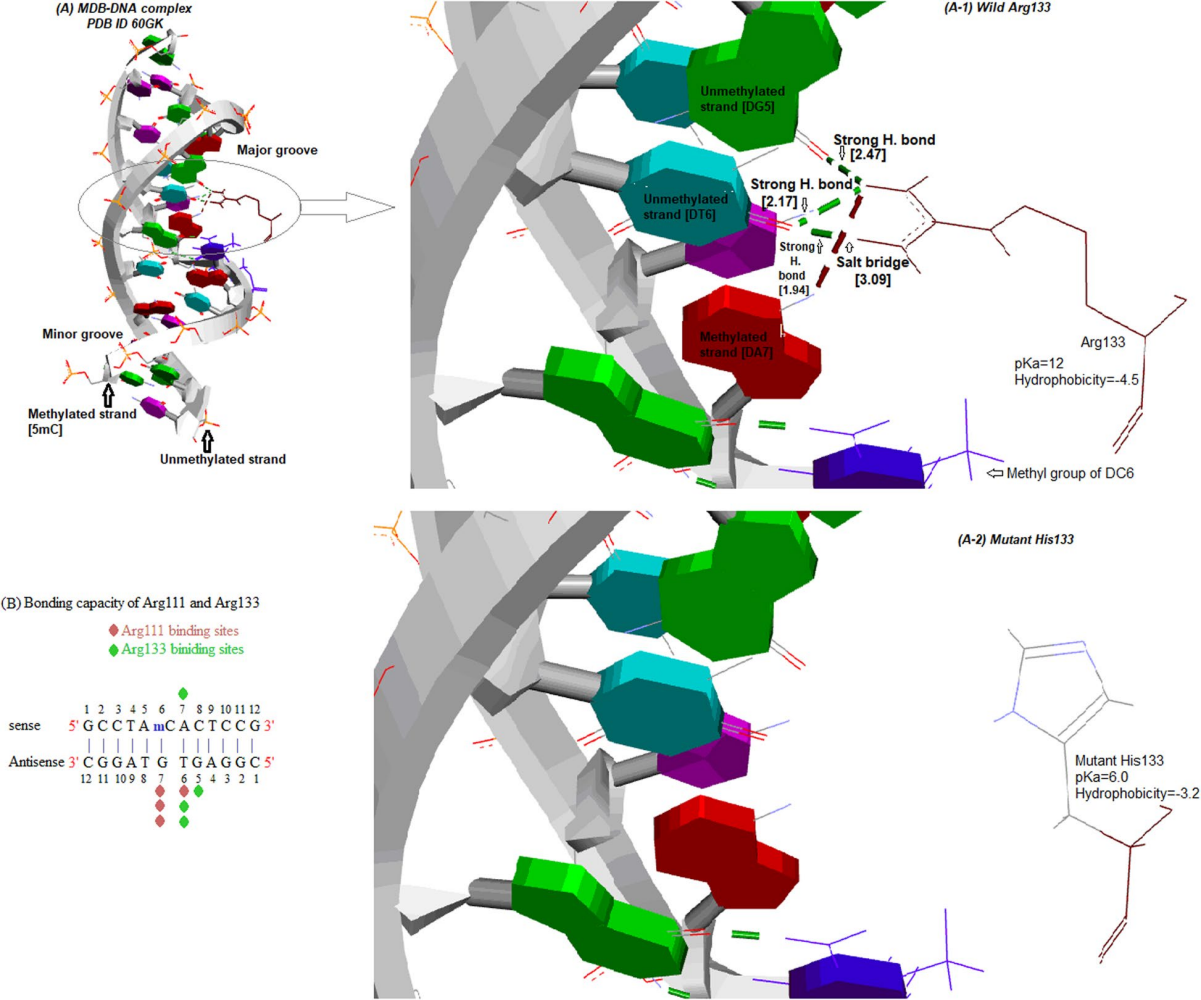
Molecular docking of R133H showed differential capacity bonding between arginine and histidine residues. **A** The major groove of DNA targeting Arg133; A-1, the capacity bonding of the wild Arg133; A-2, the capacity bonding of the mutant His133; **B** the differential capacity bonding between Arg111 and Arg133

It is noteworthy that the significant ∆∆G of D121A was between MDB and the unmethylated DNA strand only; however, the significant ∆∆G of R133H was between MDB and both DNA strands. Interestingly, extended docking analysis succeeded to raise reasonable explanations for such observation (i) as previously mentioned, D121A resulted in lower rigidity of Arg111 and Arg133 which mostly target 5*′*-GTG-3*′* trinucleotide sequence on the unmethylated DNA strand; (ii) R133H disrupt an essential salt bridge with the deoxyadenosine in the context of 5*′*-mCAC-3*′* as shown in Fig. 3.

Importantly, the methyl group of the deoxycytidine within the 5*′*-mCAC-3*′* sequence is oriented toward the main chain of both wild R133 and mutant H133 indicating that residue 133 plays a vital role in the formation of MDB-DNA molecular pocket as shown in Fig. 3.

Finally, the current bonding capacity of the two arginine fingers, Arg111 and Arg133, and Asp121 might point out that the 5′-GTG-3′ sequence is a dominant driver in MBD-DNA binding and the unmethylated DNA strand has a potential stabilizing role in the interaction between MDB and methylated DNA.

### UCC → UAC change of S359Y variant marginally decreases the thermal stability of MeCP2 mRNA

**Fig. 4:**
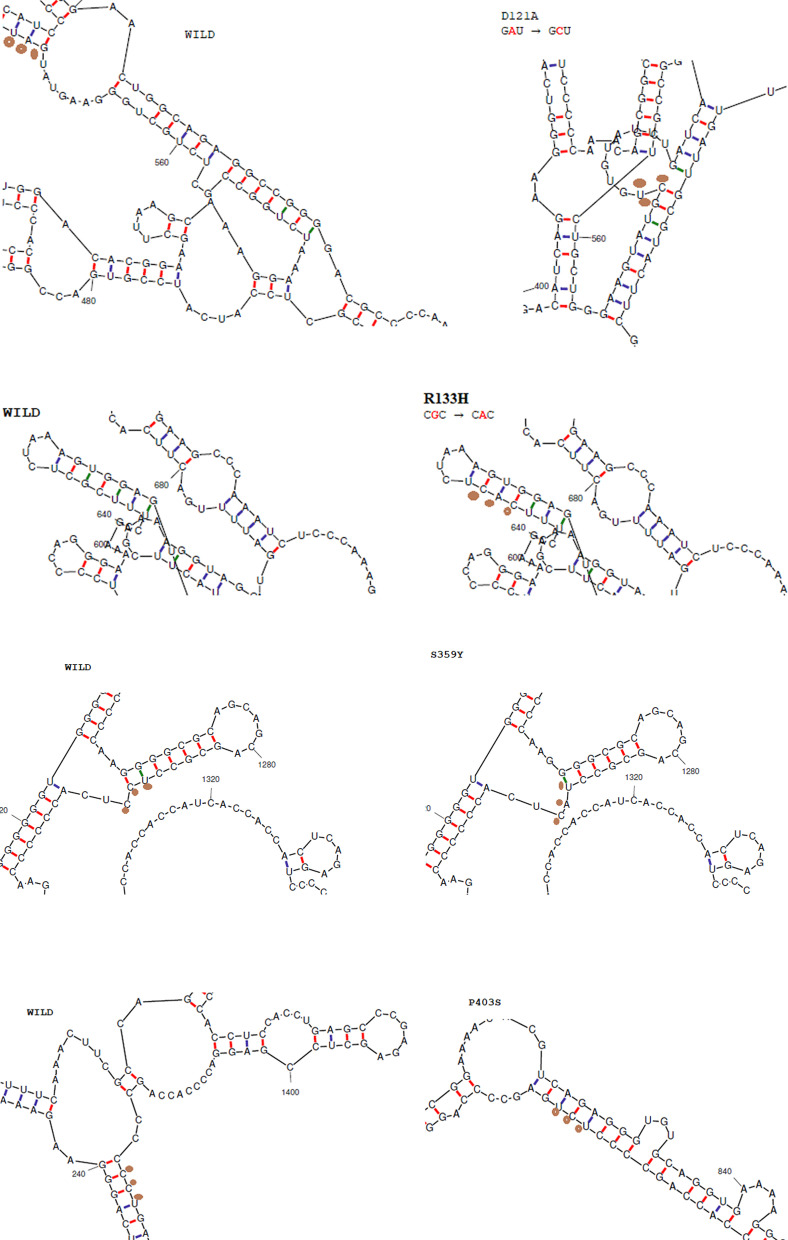
Differences in mRNA folding between wild and mutant alleles

By using the mFold server that applies the Vienna RNA folding procedure taken from Zuker’s optimal RNA folding algorithm, we identified the effect of the detected variants on mRNA folding compared to the reference sequence. The differences in mRNA structure and its thermal stability (minimum free energy) showed a marginal effect of the S359Y variant on mRNA stability. Also, faulty mRNA folding due to D121A and P403S variants was observed with slightly increased mRNA stability as shown in Fig. 4.

### Altered MeCP2 phosphorylation capacity due to extra S403 phosphorylation site

P403S showed altered positional phosphorylation. Wild proline residue is unable to attach the phosphoryl group. On the opposite side, GPS v5.0 software postulated that mutant serine residue is ready to attach the phosphoryl group creating a kinase-specific binding site. The same software was also used to investigate possible binding kinases. Importantly, GPS v5.0 output is restricted to kinases that are specific to the variant flanking sequence (around 10 amino acids). The output is then subjected to a data mining approach to identify kinases expressed in the cerebellum and cerebella tissues; we found that both CLK2 and TTBK1 can transfer the phosphoryl group to this position as shown in Table 6. However, possible alternative behavior of MeCP2 due to the new phosphorylation pattern cannot be detected.

**Table 6:** Possible kinases for the mutant S403 site

Kinase	Peptide	Score	High cutoff	∆
CK1/TTBK/**TTBK1**	SEDPTSPSEPQDLSS	13.054	10.827	2.227
CMGC/CLK/**CLK2**	SEDPTSPSEPQDLSS	0.006	0.006	0

## Discussion

MECP2 mutations are the primary cause of RTT, a serious neurodevelopmental disorder affecting females. They are scattered throughout the whole gene including point mutations, small indels, and large rearrangements [19]. Missense mutations causing RTT are mainly localized to the main gene functional domains, MBD and TRD. However, some mutations outside these domains can also mediate disease progression [13]. On the other hand, few mutations with a neutral effect have been reported in the TRD such as T228S [20], G232A, and P251L [21]. Here, we used the ROC curve analysis to investigate whether different physicochemical characteristics of normal and mutant amino acids could help in expecting the mutation effect. However, no significant results were obtained. Only mutation location is a critical determinant for variant pathogenicity. Familial investigations may provide a useful tool to rule out the pathogenicity of a specific mutation. However, studying the molecular mechanisms of missense mutations represents a critical issue to identify disease-causing variants. By virtue of high-throughput sequencing techniques, there is an exponential documentation of novel gene variations. This necessitates the application of variable computational algorithms to filter out such detected variations prior to experimental validation and to investigate possible pathogenic mechanisms. In this study, 3D structure-based methods were applied to model the effects of certain missense mutations (D121A, R133H, S359Y, and P403S) on protein stability and interactions. The 3D structure of the whole protein has been not available yet in protein data bank (PDB), so in the present study, the native MeCP2 sequence, extracted from UniProt ID P51608, was submited to Phyre2 server to predict the protein structure.

Both D121A and R133H are located in the MBD and reported in patients with classical RTT. D121A is a novel mutation; however, another sequence variation at this amino acid residue, D121G, has been previously detected. R133H is a reported mutation with low frequency (0.17%). One of the most recurrent RTT mutations (R133C) also originates at this residue with a frequency of 4.52%. Substituting Arg133 with Gly or leu has also been documented, but with a very low occurrence rate (0.04 and 0.02, respectively) [4]. On RettBase, the effect of R133H is defined as unknown. The R133H mutant protein exhibited near-normal affinity to pericentromeric heterochromatin and transcriptional repressive activity [22]. Moreover, R133H containing MBD displayed similar folding stabilities to the wild type MBD [23].

Several in vitro studies demonstrated that many missense mutations within the MBD can significantly reduce the affinity of MeCP2 to bind methylated DNA [24–27]. In particular, Arg111, totally conserved among members of the MBD family, plays a crucial role in protein binding to methylated DNA, and its mutation results in MBD without any detectable affinity for DNA [25, 28]. In the current study, we concluded that Asp121 interacts with Arg111 and Arg133 orientating the latter side chains and enabling their contact with DNA. D121A leads to increased conformational plasticity of Arg111 and Arg133 dramatically affecting their interaction with DNA. Hence, the current results point to the indirect pathogenic mechanism for D121A through its effect on Arg111 and Arg133 orientation. It is noteworthy that Lei et al. also stated that Asp121 has a potential function in the rigidification of Arg111 [29].

The mutation R133H led to the decreased affinity of MBD to methylated DNA (∆∆G=1.26). In consistence, Yang and colleagues reported that R133H decreased MBD affinity for mC over 12-fold and for C less than 2-fold [23]. In the current study, the direct pathogenic mechanism of R133H has been illustrated. As arginine is more basic (pKa=12) than histidine (pKa=6), it can form a salt bridge with DNA. This difference in the binding capacity between arginine and histidine resulted in decreased MBD affinity for DNA. In this context, it was previously reported that Arg133 is the most critical residue in DNA binding, and its mutant forms led to diminished binding affinity to methylated DNA as measured by gel mobility shift assays and structure crystallization [23, 29, 30].

Our 6OGK post-molecular analysis showed that 5′-GTG-3′ trinucleotide on the unmethylated DNA strand is the main target of Arg133 and Arg111 demonstrating the potential role of the unmethylated strand in MBD-DNA interaction. Interestingly, a ChiP-seq analysis has revealed that the percentage of native GC is more determinant for MeCP2 distribution than methylated CG dinucleotides and MeCP2 binds with methylated non-CG motifs such as mCAC found in the brain [31]. Also, crystal structures exhibited that Arg111 and Arg133 residues mainly bind to GTG trinucleotides on the unmethylated DNA strand, but 5′-mC on the complementary strand is not essential for their interaction [29].

On the other hand, both S359Y and P403S are mutations in the CTD. There was a general conception that missense mutations in the CTD have a benign effect. However, some missense mutations in CTD have been also defined as pathogenic or likely pathogenic in ClinVar (see Supplementary table 1). Moreover, it has been demonstrated that the Rett-like phenotype can be originated in mice due to specific missense mutation (P322L) in CTD [13]. Importantly, there is no difference between Pro and Leu in charge, polarity, and hydrocarbon type denoting for the minimal effect of physicochemical properties of normal and mutant residues in determining mutation pathogenicity and emphasizing the necessity for studying the molecular mechanisms of missense mutations.

S359Y is detected in association with one of the most common RTT mutations, R168X in a girl with typical RTT. In fact, more than one pathogenic mutation has been already identified in some cases with RTT [4]. Therefore, it was required to explore the possible effect of this novel variation. However, we failed to report any pathogenic effect related to S359Y denoting that disease progression in that patient is mainly mediated by R168X.

The P403S variant converts the non-phosphorylated proline residue into serine, which might provide a new phosphorylated site that can be acquired. Importantly, it was reported that most phosphorylated serine signature of MECP2 is located in its CTD [26].

Mellén et al. hypothesized that post-translational modification (PTM) of MeCP2 domains might affect the protein DNA binding capacity and its substrate specificity [32]. In particular, it is expected that mutations at activity-dependent phosphorylation sites whether inside or outside MBD impair DNA binding [33]. Mice carrying the S80A mutation displayed very mild RTT-like symptoms [34]. Moreover, mice with alanine at Ser421 and Ser424 (in CTD) were associated with a gain of function effect [34, 35]. Also, it is noteworthy that the faulty phosphorylation pattern of MeCP2 whether inside or outside MBD can directly interfere with neuronal plasticity [36]. Both Zhou et al. and Tao et al. found that dephosphorylation of S80 and phosphorylation at S421 by CamKII kinase impair neuronal activity, dendritic growth, and synaptic connection development within the cerebral cortex [34, 37]. Significantly, no MECP2 missense mutations have been reported yet at the activity-dependent phosphorylation sites. It is noteworthy that according to data provided from patient guardians, a developmental delay might have started before age of 6 months. This might suggest that this girl is a case of congenital RTT, rather than classical RTT. A congenital variant is the most disease severe form, with onset of clinical features during the first 3 months of life.

Here, we explored that both CLK2 and TTBK1 are potential co-expressed kinases that can transfer the phosphoryl group to this mutant serine residue in the cerebellum and cerebella tissues. However, we were unable to define the region containing Ser403 as recognition or binding motif for proceeding targets. Therefore, it is more likely that mutant protein has no alternative behavior; however, a confirmatory experimental analysis may be still required, as bioinformatics algorithms have limited prediction toward this issue.

## Conclusion

In conclusion, the series of prediction tools employed in the current research can define both D121A and R133H as MECP2 pathogenic variations. This confirms that affected females are patients with RTT. However, S359Y and P403S are considered benign and likely benign variants, respectively. Hence, disease progression in their patients is most likely to be mediated by other genetic variations. On the other hand, this work may provide a useful approach that can be applied to investigate the effect of other MECP2 variants in MBD and CTD and also to explore the possible mechanisms of pathogenic mutations lying in these protein domains. A main limitation is that computational tools might be unable to provide conclusive effect if the mutated residue can confer kinase binding site which in turn might bias the protein DNA binding affinity. On the other hand, we greatly recommend studying the pathogenic effect of D121A and R133H in neuronal and non-neuronal tissues to explore possible variability in DNA interactions among different tissues.

## Supplementary Information

**Additional file 1: Supplementary Table 1.** Mutations included in the ROC curve analysis.

**Additional file 2: Supplementary Table 2.** Clinical manifestations and neuroimaging of patients.

**Additional file 3: Supplementary Table 3.** Investigating the effect of MeCP2 missense mutations based on their physicochemical characteristics of wild type and mutant amino acids.

## Data Availability

The datasets used and/or analyzed during the current study are available from the corresponding author on reasonable request.

## Abbreviations

AUC: Area under the curve
CTD: C-terminal region
MBD: Methylated DNA binding domain
MECP2: Methyl CpG binding protein 2
NLS: Nuclear localization signal
NTD: N-terminal domain
ROC: Receiver operating characteristic
RTT: Rett syndrome
TRD: Transcriptional repression domain
XCL: X-chromosome inactivation
