# Supplementary material for: Variable predicted pathogenic mechanisms for novel MECP2 variants in RTT patients
Source: J Genet Eng Biotechnol. 2022 Mar 11;20:44. doi: 10.1186/s43141-022-00305-8 (PMC8917248; doi:10.1186/s43141-022-00305-8)
Supplement: Supplementary file 1 — Additional file 1: Supplementary Table 1. Mutations included in the ROC curve analysis. [file 43141_2022_305_MOESM1_ESM.docx]

**Supplementary table 1:** Mutations included in the ROC curve analysis.

| **Mutation** | **Effect** | **Polarity** | **Charge** | **Hydrocarbon type** | **Location** |
| --- | --- | --- | --- | --- | --- |
| M1L | 1 | 1 | 1 | 1 | 1 |
| M1V | 1 | 1 | 1 | 1 | 1 |
| A2V | 1 | 1 | 1 | 1 | 1 |
| L100V | 1 | 1 | 1 | 1 | 2 |
| P101L | 1 | 1 | 1 | 1 | 2 |
| P101R | 1 | 2 | 2 | 1 | 2 |
| P101H | 1 | 2 | 2 | 2 | 2 |
| P101S | 1 | 2 | 1 | 1 | 2 |
| R106Q | 1 | 1 | 3 | 1 | 2 |
| R111K | 1 | 1 | 1 | 1 | 2 |
| D121A* | 1 | 2 | 5 | 1 | 2 |
| V122M | 1 | 1 | 1 | 1 | 2 |
| P127L | 1 | 1 | 1 | 1 | 2 |
| R133C | 1 | 1 | 3 | 1 | 2 |
| R133H* | 1 | 1 | 1 | 2 | 2 |
| S134F | 1 | 2 | 1 | 2 | 2 |
| K135E | 1 | 1 | 4 | 1 | 2 |
| P152A | 1 | 1 | 1 | 1 | 2 |
| F155S | 1 | 2 | 1 | 3 | 2 |
| D156E | 1 | 1 | 1 | 1 | 2 |
| T158A | 1 | 2 | 1 | 1 | 2 |
| R162G | 1 | 2 | 3 | 1 | 2 |
| R190H | 1 | 1 | 1 | 2 | 3 |
| P217L | 1 | 1 | 1 | 1 | 4 |
| P225L | 1 | 1 | 1 | 1 | 4 |
| P225R | 1 | 2 | 2 | 1 | 4 |
| P225T | 1 | 2 | 1 | 1 | 4 |
| P302R | 1 | 2 | 2 | 1 | 4 |
| R306C | 1 | 1 | 3 | 1 | 4 |
| P322L | 1 | 1 | 1 | 1 | 5 |
| P322S | 1 | 2 | 1 | 1 | 5 |
| S346R | 1 | 1 | 2 | 1 | 5 |
| T196S | 2 | 1 | 1 | 1 | 3 |
| T203M | 2 | 2 | 1 | 1 | 3 |
| T228S | 2 | 1 | 1 | 1 | 4 |
| G232A | 2 | 1 | 1 | 1 | 4 |
| A278T | 2 | 2 | 1 | 1 | 4 |
| V288M | 2 | 1 | 1 | 1 | 4 |
| S359Y* | 2 | 1 | 1 | 2 | 5 |
| P362A | 2 | 1 | 1 | 1 | 5 |
| P385H | 2 | 2 | 2 | 2 | 5 |
| P403S* | 2 | 2 | 1 | 1 | 5 |
| G428S | 2 | 2 | 1 | 1 | 5 |
| A447T | 2 | 2 | 1 | 1 | 5 |
| R458H | 2 | 1 | 1 | 2 | 5 |

Effect: 1, pathogenic or likely pathogenic; 2, benign or likely benign

Polarity: 1, polar to Polar or nonpolar to nonpolar exchange; 2, polar to nonpolar or nonpolar to polar exchange Charge: 1, no change in charge; 2, neutral to +ve; 3, +ve to neutral; 4, +ve to –ve; 5, -ve to neutral

Hydrocarbon type: 1, aliphatic or polar to aliphatic or polar; 2, aliphatic or polar to aromatic; 3, aromatic to aliphatic or polar

Location: 1, NTD; 2, MBD; 3, ID; 4, TRD; 5, CTD

* Variants studied in the article.
