# Supplementary material for: Variable predicted pathogenic mechanisms for novel MECP2 variants in RTT patients
Source: J Genet Eng Biotechnol. 2022 Mar 11;20:44. doi: 10.1186/s43141-022-00305-8 (PMC8917248; doi:10.1186/s43141-022-00305-8)
Supplement: Supplementary file 2 — Additional file 2: Supplementary Table 2. Clinical manifestations and neuroimaging of patients. [file 43141_2022_305_MOESM2_ESM.docx]

**Supplementary table 2:** clinical manifestations and neuroimaging of patients.

| **Clinical data** | **Patient with D121A** | **Patient with R133H** | **Patient with S359Y combined with R168X** | **Patient with P403S** |
| --- | --- | --- | --- | --- |
| **Age at diagnosis** (years) | 3 6/12 | 2 6/12 | 1 4/12 | 2 |
| **Age at onset** (months) | 12 | 16 | 12 | not provided* |
| **Loss of acquired purposeful hand skills** | + | + | + | + |
| **Loss of acquired spoken language** | + | - | + | + |
| **Microcephaly** (SD) | -1.37 | -1.6 | -2.4 | -2.7 |
| **Growth retardation** | - | - | - | + |
| **Ambulation** | + | - | - | - |
| **Stereotypic hand movements** | + | + | + | + |
| **Breathing disturbances when awake** | + | + | - | - |
| **Bruxism when awake** | - | - | + | + |
| **Impaired sleep pattern** | + | + | + | - |
| **Inappropriate laughing/screaming spells** | + | _ | + | + |
| **Diminished response to pain** | + | _ | + | + |
| **Intense eye communication** | + | + | + | _ |
| **Scoliosis/kyphosis** | - | - | - | - |
| **Abnormal Muscle tone** | hypotonia | hypotonia | hypotonia | hypotonia |
| **Small hands and feet** | - | + | - | - |
| **Peripheral vasomotor disturbances** | - | - | - | - |
| **Constipation and bowel disturbances** | + | - | - | - |
| **Epilepsy** | - | - | + | - |
| **MRI changes:** | cortical atrophy, minimal deep white matter changes, thin corpus callosum. | - | cortical atrophy | dilated Lateral ventricles, cortical atrophy, thin corpus callosum |

*patient guardians can’t ensure when developmental delay have be already started. It might be congenital.
