# Supplementary material for: Variable predicted pathogenic mechanisms for novel MECP2 variants in RTT patients
Source: J Genet Eng Biotechnol. 2022 Mar 11;20:44. doi: 10.1186/s43141-022-00305-8 (PMC8917248; doi:10.1186/s43141-022-00305-8)
Supplement: Supplementary file 3 — Additional file 3: Supplementary Table 3. Investigating the effect of MeCP2 missense mutations based on their physicochemical characteristics of wild type and mutant amino acids. [file 43141_2022_305_MOESM3_ESM.docx]

**Supplementary** **table 3**: Investigating the effect of MeCP2 missense mutations based on their physicochemical characteristics of wild type and mutant amino acids.

| **Variable(s)** | **AUC** | **P-value** |
| --- | --- | --- |
| Location | 0.871 | 0.000 |
| Polarity | 0.543 | 0.652 |
| Charge | 0.376 | 0.197 |
| Hydrocarbon type | 0.518 | 0.851 |
| Location + Polarity | 0.871 | 0.000 |
| Location + Charge | 0.907 | 0.000 |
| Location + Hydrocarbon type | 0.880 | 0.000 |
| Location + Polarity + Charge | 0.909 | 0.000 |
| Location + Polarity + Hydrocarbon type | 0.879 | 0.000 |
| Location + Charge + Hydrocarbon type | 0.911 | 0.000 |
| Location + Polarity + Charge + Hydrocarbon type | 0.912 | 0.000 |

Analysis was applied for mutations with known clinical significance on ClinVar including our studied variants. AUC, area under the curve.
